# Using Photographs to Bring Dignity to Patients and Help Clinicians Find Meaning and Joy in Work

**DOI:** 10.1089/jpm.2022.0161

**Published:** 2023-01-27

**Authors:** Ali Mendelson, Bryce Bandfield, Julie Hevezi, Jason Gable, Judy E. Davidson, Gary Buckholz

**Affiliations:** ^1^ Kaiser Permanente Washington, Seattle, Washington, USA.; ^2^ UC San Diego Health, San Diego, California, USA.

**Keywords:** burnout, compassion, dignity, empathy, goals of care, physical appearance

## Abstract

**Background:**

The importance of dignity in health care is well described, yet limited interventions exist to improve dignity, particularly patient-driven interventions.

**Objectives:**

To test the hypothesis that patient-selected photographs at the bedside would impact patients' sense of dignity and clinicians' sense of meaningful work, stimulate conversation between patients and clinicians, and serve as a visual and patient-driven complement to the Patient Dignity Question (PDQ).

**Methods:**

Patients admitted to three units at an academic medical center displayed photographs above their head of bed and were interviewed for this study. We used thematic content analysis to compare themes extracted from patient interviews, the PDQ, and clinician surveys.

**Results:**

Eight themes emerged from patient interviews (*n* = 19): conveying goals, joy, capturing the patient's spirit, faith and spirituality, sense of belonging, physical appearance and health, stimulating conversation and meaningful connections, and humanizing the patient. The same themes emerged from the PDQ, with the exception of physical appearance and health. Notably, analysis of the clinician surveys (*n* = 40) yielded six similar themes: conveying goals, joy, stimulating conversation and meaningful connections, humanizing the patient, meaningful work, and compassion and empathy.

**Conclusions:**

Patient-selected photographs at the bedside impact both patients and clinicians by stimulating conversation and meaningful connections, humanizing patients, and fostering meaning and joy in work. Photographs and the PDQ provide a similar window into personhood, thereby supporting the use of a photograph as a visual and patient-driven complement to the PDQ.

## Introduction

Dignity is a universal human quality and basic right.^1^ Dignity is generally defined as the “quality or state of being worthy, honored, or esteemed,” yet it is a complex, multidimensional concept that is influenced by culture and varies between individuals.^1,2^

In health care, respect for dignity appears within the first principle of the American Medical Association and American Nurses Association codes of ethics.^3,4^ Palliative care evolved as a unique subspecialty dedicated to supporting dignity by addressing physical, psychological, social, and spiritual distress and focusing on what matters most to patients and their families.^1^ Research has shown that recognizing and helping to support a patient's unique sense of personhood is foundational to preserving their dignity and can improve communication and perceived care quality.^5–7^ Yet there is a striking paucity of research on interventions designed to promote personhood and enhance dignity.^8^

Currently, the most established tool is dignity therapy. Dignity therapy was developed by Harvey Chochinov to improve dignity at the end of life by addressing the psychosocial and existential distress experienced by dying patients.^9–11^ The Patient Dignity Question (PDQ) is similarly based on the dignity model but encourages all clinicians to ask their patients, “What do I need to know about you as a person to give you the best care possible?”^12^ The PDQ is widely accepted by patients and families, affects how clinicians see, feel about, and care for their patients, and has a positive impact on patient-centered and empathetic care.^6,12,13^ The expectation is not that clinicians ask the PDQ during every patient encounter but that they learn to see each patient as an individual with a unique sense of personhood rather than a disease or checkbox on a to-do list.

The reliance on clinicians to ask the PDQ represents an opportunity to consider the role of patient-driven interventions, and there is mounting evidence to support the use of a photograph as a similar window into personhood. Harvey Chochinov's work was inspired by a bedside photograph, and it has been our experience that hospitalized patients often have photographs or other significant mementos at their bedside. In a feasibility study exploring the use of photographs as a novel tool to improve dignity, we have previously shown that that bedside photographs stimulated conversations with providers and improved the hospital experience among patients admitted to an inpatient neurology unit.^14^

Hubbard et al found that displaying a photograph at the bedside in a rehabilitation ward personalized the environment, promoted patients' self-identity, and improved connections and communication between patients, caregivers, and staff.^5^ They noted overlap between the themes identified in their study and those in prior dignity research, supporting a need for future studies exploring the role of a photograph as a dignity-enhancing tool.

There is also evidence that bedside photographs impact clinicians. In the Intensive Care Unit (ICU), bedside photographs serve as a “landmark bringing hope” and help clinicians relate to patients as people.^15,16^ Similarly, in a burn ICU, preinjury photographs of patients have been shown to positively impact the quality of nursing care and the ability of nurses to meet the psychological needs of their patients.^17^

The impact on clinicians is acutely relevant because burnout is increasingly prevalent and directly affects wellness and job retention, as well as patient care and satisfaction.^18–20^ An inherent sense of meaningful work and compassion can protect clinicians against burnout.^21–23^ Unfortunately, clinicians are spending increasing amounts of time in nondirect patient care, which is associated with greater job dissatisfaction and increased risk of burnout.^24,25^ This study was funded by the Back to Bedside program, which was developed by the Accreditation Council for Graduate Medical Education with the goal of creating and supporting functional improvements within medical learning environments, particularly those that foster direct bedside engagement with patients and families and bring meaning and joy to trainees' work.^23^

Our hypothesis was that patient-selected photographs at the bedside would impact patients' sense of dignity and clinicians' sense of meaningful work, stimulate conversation between patients and clinicians, and serve as a visual and patient-driven complement to the PDQ.

## Materials and Methods

This Institutional Review Board-approved (No. 180167) qualitative research study was conducted with data gathered between February 2019 and June 2020 in three units at an academic medical center in Southern California: cardiology/medical/surgical progressive care unit (PCU), medical/surgical ICU, and oncology/palliative care PCU. The study had two target populations: patients (referring to patients or their surrogates) and clinicians.

### Patients

Flyers were distributed to patients admitted to the participating units. The flyers invited patients to choose a single photograph representing how they want to be seen by their health care team. There was no guideline or restriction as to what photographs were allowed. The photographs were displayed above patients' head of the bed as standard of care. All patients were screened by a charge nurse for eligibility after their photograph was displayed for >24 hours. If interested, they were consented by a member of the research team. Non-English-speaking patients were not eligible to participate in the study.

Patients were also excluded if they declined, got discharged from the hospital, got transferred to a nonparticipating unit, died before being interviewed, or were critically ill and unavailable to be interviewed. Patients were not excluded based on age, gender, or ethnic background. A surrogate decision maker could consent and participate on a patient's behalf. Informed and media usage consents were obtained. The aim was to interview a convenience sample of 15–30 patients.

Interviews consisted of three questions exploring why patients chose a specific photograph, if it was helpful to have the photograph posted on their wall, and if it improved their sense of dignity. Patients were also asked the PDQ. The interviews were conducted by three members of the research team (J.G., J.H., and B.B.) within their respective units following a training session and trial interview with another member of the research team (A.M.). Face-to-face interviews were recorded on audio devices, except for one subject who was unable to speak and provided written responses.

### Clinicians

Post-intervention clinician surveys were sent by e-mail. The open-ended responses to two items exploring the impact of the photographs on clinicians were the focus of this study: (1) How helpful was it to see photographs of patients representing how they want to be seen by providers? (2) Seeing photographs of patients representing how they want to be seen by providers helped me recognize the meaning in my work.

Clinicians were identified by contacting the nurse managers and heads of the departments whose physicians work in or rotate through the participating units. The managers or department heads either forwarded the e-mail or provided a contact list for distribution. The aim was to survey all clinicians who interacted with patients in the three participating units.

### Data analysis

Patient interviews and open-ended clinician comments were analyzed using thematic content analysis.^26^ Quotes are included verbatim and have not been edited for grammar.

Trustworthiness was established through analyst triangulation. A primary researcher (B.B., J.G., or J.H.) was assigned to transcribe the transcripts of interviews conducted within their own unit. Each transcript was reviewed by a secondary researcher (A.M.). Four researchers (A.M., B.B., J.G., and J.H.) independently immersed themselves in the dataset from each individual unit and then came together to achieve consensus on codes and associated themes. Data from the three units were then pooled, and the research team met iteratively to compare and contrast findings through which consensus was built on a final list of codes and themes.

Dependability was established by maintaining an audit trail. Logs were maintained of patient interviews. Data and data analyses were maintained on a shared drive. Confirmability was established by having two investigators not engaged in the initial coding (J.E.D. and G.B.) read through the final reports and agree that the passages linked to themes were accurately assigned. Transferability was established by collecting data from three different hospital units, analyzed separately and then together. Authenticity was enhanced by gathering data from the perspectives of both patients and clinicians. Investigators had a wide range of backgrounds (PCU, ICU, palliative care, nurse, physician, and researcher) and represented several generations, providing opportunities for bias-checking.^27^

## Results

### Demographics

**FIG. 1.**
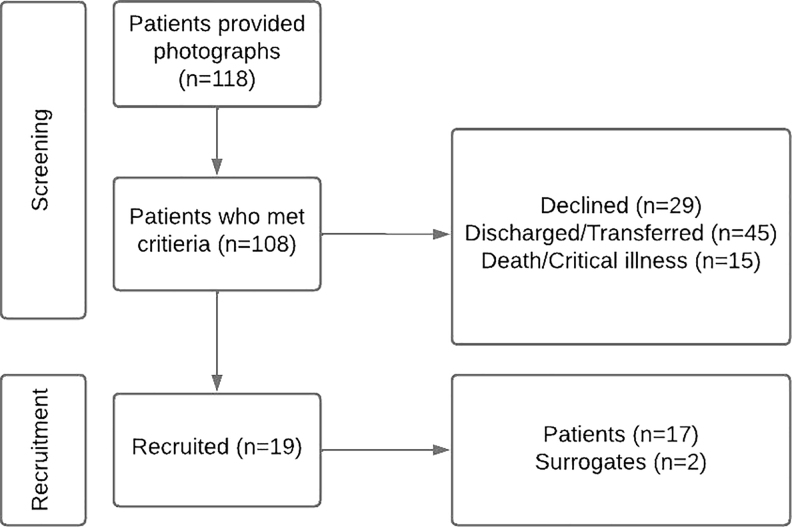
Patient recruitment and demographics.

**FIG. 2.**
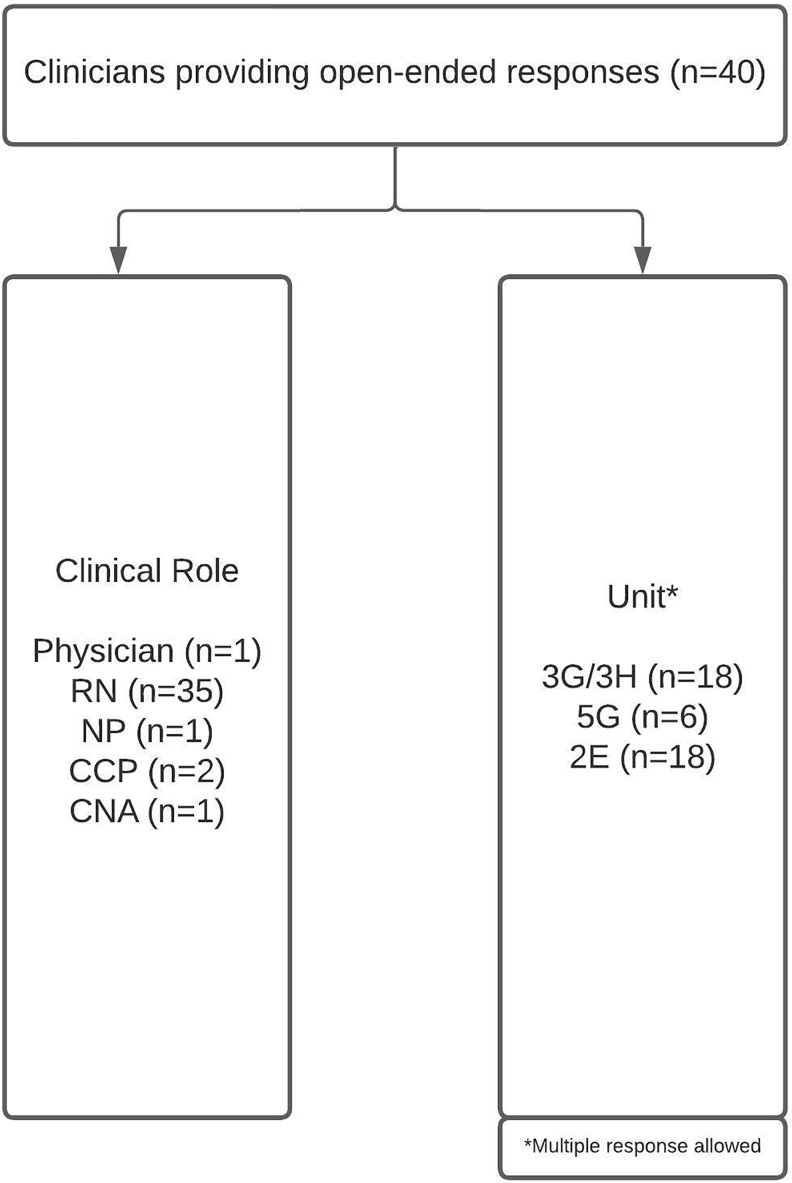
Clinician demographics. 2E, cardiology/medical/surgical progressive care unit; 3G/3H, medical/surgical intensive care unit; 5G, oncology/palliative care progressive care unit; CCP, clinical care partner; CNA, certified nursing assistant; NP, nurse practitioner; RN, registered nurse.

Nineteen patients participated in the study (Fig. 1). Forty clinicians, of which 35 were nurses, provided 63 open-ended responses in the post-intervention survey (Fig. 2).

### Patients

Eight themes emerged from the patient interviews. The same themes emerged from the PDQ, with the exception of physical appearance and health (Table 1).

**Table 1.** Themes and Representative Quotes: Patient Perspective and the Patient Dignity Question

Theme	Representative quotes (photographs)	Representative quotes (PDQ)
Conveying goals Goals and hope for the future	They look at that, and they look at him, and they see what they have to get him to. That's my world, that's my son, and if it wasn't for him, I'd be dead, and that's why I look at it every day. It keeps me fighting and fighting. He's my reason for living. He's my reason to fight.	Survival. I want to live a little longer. I want to do things. I do think that if I might know more about them and they might know more about me or my life and what I've been doing and what I would like to continue doing then I perhaps would have improved care.
Joy Sharing sources of joy (i.e., family, art, hiking) Sharing happy memories Humor	When someone asks about it, it just brings so much happiness and joy. I chose the photo because I remember feeling happy, strong, healthy, and very much myself that day. It was the day of my daughter ^***^'s high school graduation. The sun was shining, and it was a beautiful day. It keeps my spirits alive. That's for sure. It gives me hope. It gives me joy. I chose this photograph because it's one of the happier moments that I had … I was so excited to be picked to go up on stage at the Price is Right … it was a day of real excitement for me. Playing guitar is a major part of my life. It's what I do to relax and calm myself … My wife picked out the photograph because it most reminds her of where my happy place is. I chose this photograph because it showed me living. I went to Subway, the sub store, this particular day … and I wore my onesie. And I wanted people to see I'm alive, I'm happy, I'm full of fun.	He's a Harley rider, he loves dogs, and he loves living life.
Capturing the patient's spirit “I want to show …” (i.e., resilience, gratitude, love for life)	It shows my workers, the people taking care of me … that I'm full of life and happiness. They comment on it all the time, that I'm full of fun. And they're just amazed. I chose that photo because ^***^ is very optimistic, and it shows that with his thumbs up. And also he loves nature, he loves plants … It was such a beautiful day with the rainbow. It just sort of represents his spirit.	We want to live. Look at my photo, and you'll see … I've never felt anything other than I want to live. I haven't wallowed in my own sadness. I've just tried to be happy, and that's what I want to pass off to everybody else. Just be happy. And that's what I want others to know about me. Be happy. I guess if anything, it would be I'm a strong person. … I've been by myself for a lot of years, so I'm not … weak in any means. I'm strong … so I guess I wouldn't want to be treated as weak … I am a strong person, so I would rather be treated that way.
Humanizing the patient “I want to be seen as …”	It shows that the providers, the staff, the hospital, the program, is interested in the individual as an individual, rather than a patient. I do hope that if a provider sees the photograph of me that it reminds them that I am more of a person than a sick body. Maybe it will inspire to not only operate at a mind/brain level but also through the heart. So yes, I would say the photo posted on the wall does improve my sense of dignity. The custodians, or the nurses, or even the doctors come in, and they see me sitting here in a gown, with oxygen and everything on me and all the wires and stuff. They look up at the picture, they know there is another side of me, you know there's … a normal side of me, so I think it really helps. It helps … them understand me, and through that … they are more comfortable talking to me, which in turn makes me more comfortable with interacting with the doctor. It really humanizes the patients … I know the nurses and the doctors see people in all kinds of conditions, and it's hard to picture them normal, in a normal life, in a normal setting … The pictures that people submit, I think it helps see them in another light, and it just, it makes things more comfortable. Now I am not just the guy laying in a bed where they bring me medicines on occasion a couple times a day. It's a person that they're treating.	I think, like you said … I'm not a disease … I'm a whole person. And I think that's so important for them to see. I'm human … I've been sick since March that I know of, and in that period of time, I think the most offensive moments that I've had is where I become just a number, or I become just a you're-in-the-way kind of thing. It's important for my provider to know that I do well when I am truly heard and seen. I guess basically just that I enjoy being respected … when, when a doctor talks to me as a person and not, you know, at me, as if I don't know anything.
Faith and spirituality	It is nice to see the photo of me because it reminds me of that beautiful day and also how resilient the body is. It also reminds me of how good God is.	First thing I can think of is that they need to know is that I am a Christian, that I love the Lord, and he is my savior.
Sense of belonging “I matter to someone” (i.e., family, friends)	It makes the nursing staff and the doctors realize that they're just not a number. They really are an important person to some people. It just like shows a lot of love of her daughter, my granddaughter, you know, hugging me. I think it improves my sense of belonging … I love him being up there because people come in and they go “Oh, what a cute picture,” so I think it makes me feel, you know, that they care about me as a patient … it means a lot.	That my family is number one for me is very, very important for me. My children mean a lot to me, they, I mean, it's amazing the care and love that they show me. It's incredible. It takes my breath away.
Physical appearance and health	I like people seeing what I used to look like compared to what I look like now. I chose that photograph because I think it shows that I still have energy and I was enjoying life before I knew I had cancer. I look good in it.	
Stimulating conversation and meaningful connections	I've had nurses and even the doctors comment and ask about the photographs, where they were taken, what my dog's name is, and I feel that they are getting to know me a little bit better rather than just my illness. Everyone who comes into my room, even the … custodians, they look up at the picture, and it starts a conversation. And you know rather than me sitting here … and people doing things around me and us not interacting, it's fun when they come in and look at the picture and go “Oh, what happened there?.” … you know it kind of starts the conversation. And while they're working around me, you know we're, we're interacting … it makes everything a lot more comfortable. It's kind of an ice breaker. … we all say hello several times a day, are polite to people, but learning one little nugget of information about somebody that you have in common or don't allows you to have a more open relationship … It allows others to trust that person … They do care and they are going to do the best job they can for you. You've got the photograph on the wall. It says something about me that I enjoy, maybe something we have in common or not have in common. Once again, it puts together a personal touch, which I think takes it out of the mathematical equation. Now it's not just a transaction. It's a relationship, where no matter how big or how small, that interaction you have is more valuable.	I like to connect with people. I like to know their story as much as I want them to know my story.

PDQ, Patient Dignity Question.

#### Conveying goals

The photographs gave patients an opportunity to share their goals and express hope for the future. One family member noted that, “They look at that, and they look at him, and they see what they have to get him to.” For other patients, the photographs served as internal motivation, reminding them, for example, that they want to get better to meet a new grandchild or care for their son.

#### Joy

**FIG. 3.**
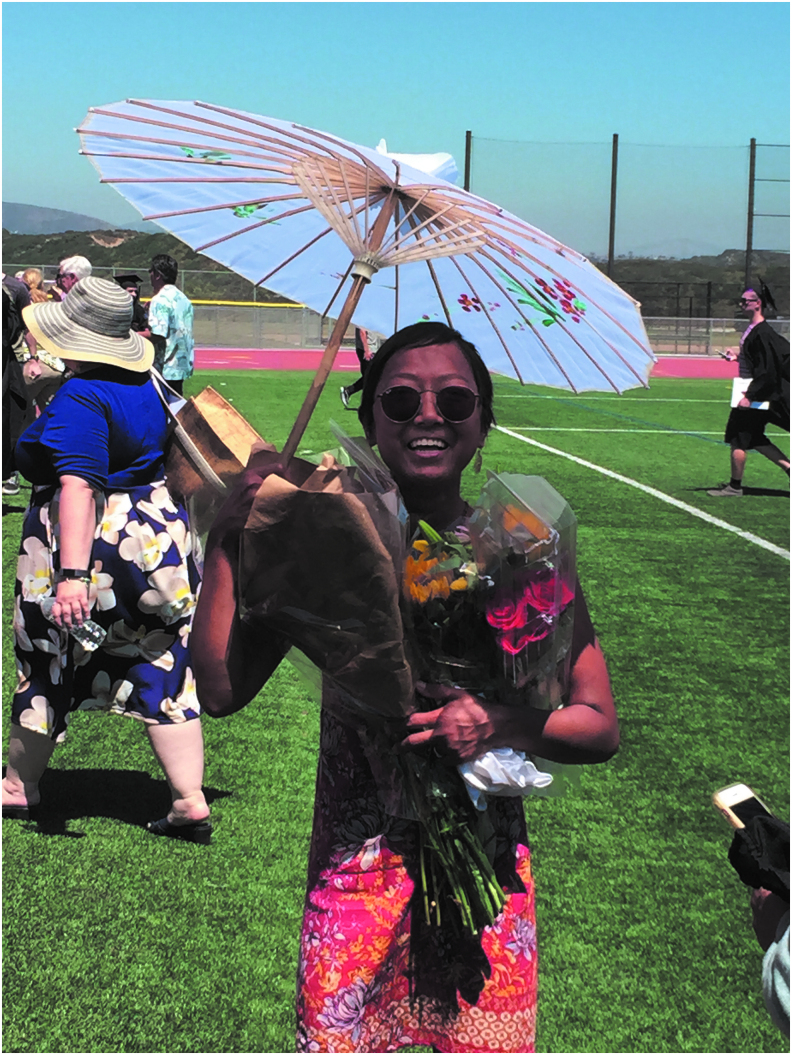
Joy: “I chose the photo because I remember feeling happy, strong, healthy, and very much myself that day. It was the day of my daughter ***'s high school graduation. The sun was shining, and it was a beautiful day.”

Patients regularly noted that the photographs brought joy to their hospitalization. Many chose photographs that portrayed happy memories or sources of joy (i.e., family, pets, and hobbies), while others chose to share their sense of humor or funny stories. As one patient explained, “I chose the photo because I remember feeling happy, strong, healthy, and very much myself that day. It was the day of my daughter ***'s high school graduation. The sun was shining, and it was a beautiful day” (Fig. 3).

#### Capturing the patient's spirit

**FIG. 4.**
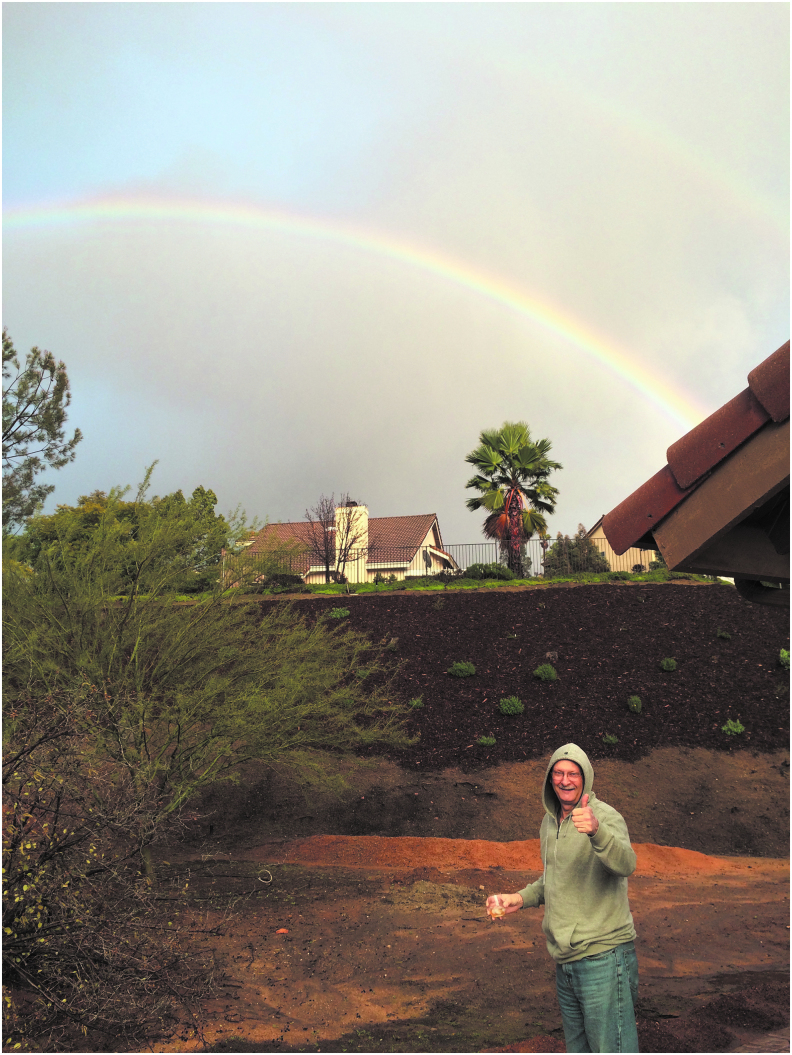
Capturing the patient's spirit: “I chose that photo because *** is very optimistic, and it shows that with his thumbs up. And also he loves nature, he loves plants … It was such a beautiful day with the rainbow. It just sort of represents his spirit.”

Photographs were often selected because they captured the patient's spirit and showed traits such as resilience, gratitude, a positive attitude, love for life, and/or individuality. As one participant explained, “I chose that photo because *** is very optimistic, and it shows that with his thumbs up. And also he loves nature, he loves plants … It was such a beautiful day with the rainbow. It just sort of represents his spirit” (Fig. 4).

#### Humanizing the patient

Patients frequently noted the impact of the photographs on personhood and described the importance of being seen as a person rather than a patient or disease. As one patient said, “Now I am not just the guy laying in a bed where they bring me medicines on occasion a couple times a day. It's a person that they're treating.”

#### Faith and spirituality

For a few patients, it was important that the photograph represents their faith or belief in something bigger, either to share their spirituality with their health care team or to serve as a personal reminder. One patient commented, “It is nice to see the photo of me because it reminds me of that beautiful day and also how resilient the body is. It also reminds me of how good God is.”

#### Sense of belonging

Several photographs included patients alongside family and friends, with the goal of showing clinicians that the patient is important to others or that there are people who rely on them because “It makes the nursing staff and the doctors realize that they're just not a number. They really are an important person to some people.” Other patients noted that the photograph improved their sense of belonging.

#### Physical appearance and health

Several patients chose photographs that portrayed their prior physical well-being or health. For example, “I like people seeing what I used to look like compared to what I look like now.”

#### Stimulating conversation and meaningful connections

**FIG. 5.**
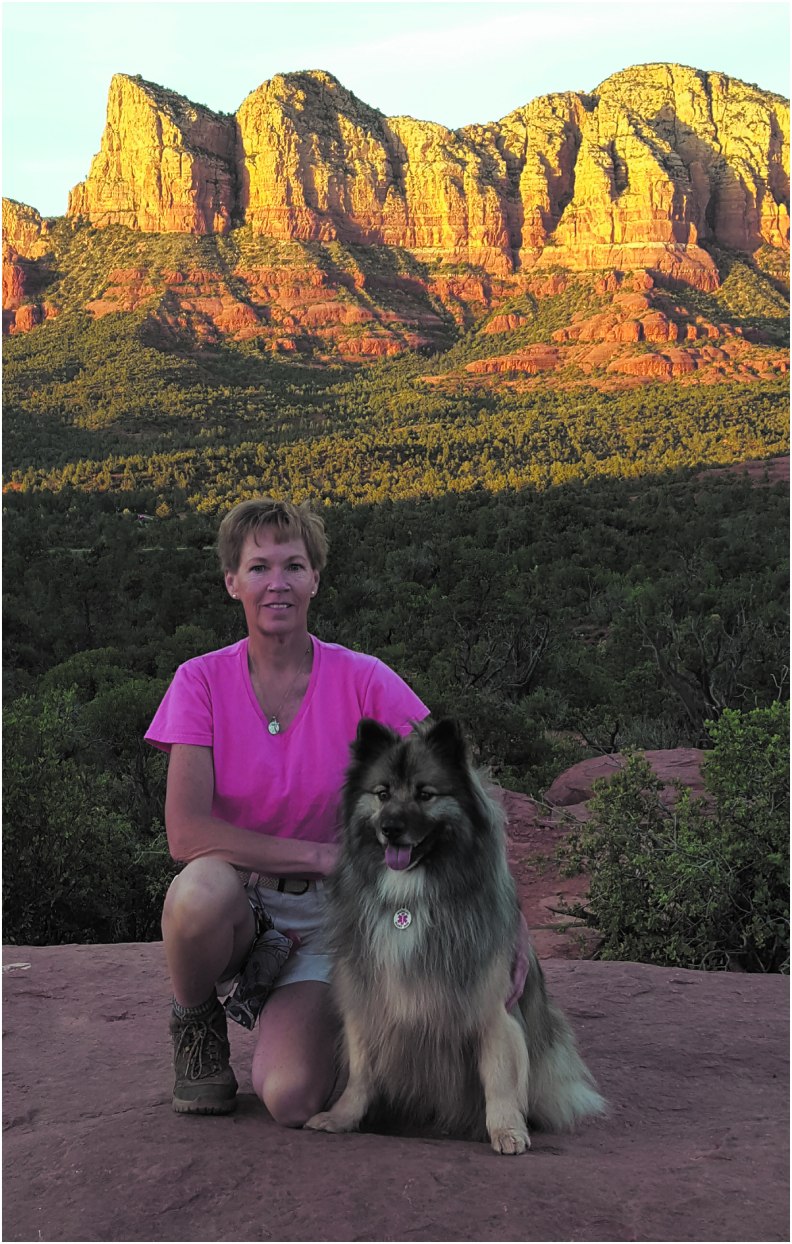
Stimulating conversation and meaningful connection: “I've had nurses and even the doctors comment and ask about the photographs, where they were taken, what my dog's name is, and I feel that they are getting to know me a little bit better rather than just my illness.”

Patients noted the importance of connecting with clinicians and found that the photographs stimulated conversation and helped forge meaningful connections. For example, “I've had nurses and even the doctors comment and ask about the photographs, where they were taken, what my dog's name is, and I feel that they are getting to know me a little bit better rather than just my illness” (Fig. 5).

### Clinicians

Six themes emerged from the clinician surveys (Table 2).

**Table 2.** Themes and Representative Quotes: Clinician Perspective

Theme	Representative quotes
Conveying goals	I want to make my patients feel if not 100% but close to what their goal is/are. It helped me to picture them in their “normal” lives and gave me hope that they can one day leave the hospital and return to doing what they enjoy. Makes you humanize the patient more and get to see them when healthy and makes you strive to get them back to [their] healthy selves. You realize that they have a high quality of life outside of the ICU and we can create an image of what we are fighting for (to get them back to that quality of life). It was a helpful reminder that the goal is not necessarily to cure everyone, but to try and get them to a point where they can return to a more normal life so they can enjoy their time.
Joy	It brings back the good memories. Its really helpful to see the families look through photos and pick out their favorite ones, and remember their loved one as healthy and vibrant. It gives family hope when all is lost. Brings joy and positivity when caring for patients.
Humanizing the patient	It helps to quickly put the patient before you in a greater context of their life. Seeing a picture of the patient outside of the hospital setting helps give clinicians a glimpse into who they are as a person. It refocuses the clinicians' minds to the fact that we are caring for someone's mother/father/husband/wife/brother/sister/aunt/uncle/friend. … All in all, we are caring for a human, seeing a picture of them helps humanize them. It helps to humanize patients and see them as someone with a life and a story. Not just a patient. It helps to remind me that a patient has been much, much, much more than the illness that is ravaging their body now.
Stimulating conversation and meaningful connections	They always look at their happiest state on the picture. Sometimes we don't even see people on the bed, sometimes we just see the cancer, pain, vomit etc. It's all tasks that we need to do. The picture is very personal and connects and encourages conversation. A way to get to know your patients before speaking to them. Great conversation piece and a method to create a connection with our patients. They served as great conversation starters. It was nice to be able to easily ask patients about their interests and family in the photographs.
Compassion and empathy	Seeing smiling faces and having a good time with friends and family before becoming sick helps empathize with patients. I had more empathy when I would get frustrated that someone was intubated for too long and care wasn't withdrawn. It helped remind me that there was someone's mom, sister, daughter, or friend behind all the tubes and wires. I came into this profession to be able to help more people. It can be very fatiguing work and tempting to lose empathy. It helps to see these photos and remember why I got into this profession and have compassion. I feel as though the photographs gave meaning to the patient and I was able to see the patient for who they are. Our cases in the ICU are sometimes very difficult and hard to not feel burnt out by them, but with the photos it helps me be a more compassionate [caregiver].
Meaningful work	I feel like I am making a difference in a life, not just another statistic. You treat a disease, you win, you lose. You treat a person, I guarantee you, you'll win, no matter what the outcome.

ICU, intensive care unit.

#### Conveying goals

Photographs helped clinicians identify and support their patients' goals by uniting the medical goals with patients' personal goals and values. As one clinician explained, “It helped me to picture them in their ‘normal’ lives and gave me hope that they can one day leave the hospital and return to doing what they enjoy.”

#### Joy

Joy was notable in several responses as a benefit for both patients and clinicians, for example, “Brings joy and positivity when caring for patients.”

#### Humanizing the patient

Clinicians frequently described the benefit of seeing patients as people, noting that, “It helps to remind me that a patient has been much, much, much more than the illness that is ravaging their body now.”

#### Stimulating conversation and meaningful connections

Clinicians also found that the photographs stimulated conversations with patients and encouraged human connection, noting that, “They always look at their happiest state on the picture. Sometimes we don't even see people on the bed, sometimes we just see the cancer, pain, vomit, etc. It's all tasks that we need to do. The picture is very personal and connects and encourages conversation.”

#### Compassion and empathy

Compassion and empathy were identified in quotes that described clinicians' responses to the photographs and/or seeing patients as people. For example, “I came into this profession to be able to help more people. It can be very fatiguing work and tempting to lose empathy. It helps to see these photos and remember why I got into this profession and have compassion.”

#### Meaningful work

Meaningful work emerged as an individual theme, arising from quotes such as “I feel like I am making a difference in a life, not just another statistic.”

## Discussion

We show that patient-selected photographs at the bedside (1) impact both patients and clinicians by stimulating conversation and meaningful connections, humanizing patients, and fostering meaning and joy in work, and (2) elicit similar themes as the PDQ. These results support the use of a photograph as a visual and, importantly, patient-driven complement to the PDQ and may inform future research and practice related to patient experience and clinician burnout.

Our study includes several strengths. This was the largest study to date looking at the impact of photographs on the dignity of inpatients and included multiple patient populations. Compared to prior studies using a single rehabilitation, neurology, or ICU, we included patients admitted to a cardiology/medical/surgical PCU, medical/surgical ICU, and oncology/palliative care PCU.^5,14,16^ Similar to studies by Mendelson and Holder and Hubbard et al, we found that photographs can stimulate conversation and encourage meaningful connections between patients and clinicians.^5,14^ We expanded on these prior studies by comparing themes extracted from patient interviews and the PDQ. Similar themes emerged, thereby demonstrating the value of a photograph as a dignity enhancing tool.

An additional strength of this study was placement of the photographs above the head of the bed to enhance viewability. This design was based on the feasibility study by Mendelson and Holder and the lack of a designated space at the bedside described in the study by Hubbard et al.^5,14^

Regarding the impact of bedside photographs on clinicians, we found that photographs can provide hope and align patient and clinician goals, similar to the findings by Andersson et al.^16^ While participants in the study by Andersson et al described both positive and negative aspects of getting closer to patients and noted that it can be emotionally difficult, although valuable, to humanize patients, our study did not reveal similar negative consequences for clinicians. This is consistent with findings by Neto et al, in which nurses felt that seeing photographs of patients had the potential to be upsetting but ultimately did not think it affected their care or made them more emotionally involved than they would have preferred.^15^ Importantly, we found that photographs helped clinicians recognize meaning in their work, which can protect against burnout.^21–23^

### Limitations

There were limitations related to both clinician and patient recruitment and retention (Figs. 1 and 2). There were no contact lists that included all clinicians working in the participating units. We instead relied on contact lists from nurse managers and heads of individual departments who work in or rotate through the units. Likely as a result, most of the responses were from nursing staff. In addition, some departments were unable to provide a contact list and forwarded the e-mail containing the consent and survey themselves, hindering our ability to track response rates.

There were also limitations related to patient recruitment and retention (Fig. 1). Patients were asked to display photographs before screening and recruitment, and non-English speakers were excluded from the study, which may have introduced selection bias. Some patients described difficulty accessing a photograph while admitted, and many had to rely on family or friends to provide one, which may have impacted the photographs selected. A potential solution to this barrier would be to encourage patients to choose a photograph as an outpatient and upload it to their electronic medical record. The photograph could then be printed and displayed upon admission to the hospital. Our experience is that, while this is an option in many electronic medical records and may even reduce medical errors, it is underused, which presents an opportunity for future studies.^28^

## Conclusion

Patient-selected photographs similarly impact patients and clinicians by stimulating conversation and meaningful connections, humanizing patients, and fostering meaning and joy in work. Both photographs and the PDQ provide a window into personhood, thereby supporting the use of a photograph as a visual and patient-driven complement to the PDQ. Future studies could investigate the impact of integrating photographs into electronic medical records on patients' sense of dignity and clinicians' sense of meaningful work in both inpatient and outpatient settings.
